# Mental health nurses experience of the introduction and practice of the Safewards model: a qualitative descriptive study

**DOI:** 10.1186/s12912-021-00554-x

**Published:** 2021-03-11

**Authors:** Heather Lee, Owen Doody, Therese Hennessy

**Affiliations:** 1 Mid-West Health Service Executive, Limerick, Ireland; 2 Department of Nursing and Midwifery, Health Research Institute, University of Limerick, Limerick, Ireland; 3 Department of Nursing and Midwifery, University of Limerick, Limerick, Ireland

**Keywords:** Acute care, Mental health, Safewards, Conflict, Containment, Ireland

## Abstract

**Background:**

A lack of safety experienced by patients and staff in acute psychiatric units is a major concern and containment methods used to manage conflict have the potential to cause harm and upset to both staff and patients. To ensure safety for all, it is highly desirable to reduce levels of conflict and containment and the Safewards model is an evidence-based model aimed at reducing conflict and containment rates by improving nurse-patient relationships and safety.

**Methods:**

The aim of this study was to explore mental health nurses’ experience of the introduction and practice of three Safewards interventions; reassurance, soft words and discharge messages. A qualitative descriptive research design utilising a purposive sample (*n* = 21) of registered psychiatric nurses (*n* = 16) and managers (*n* = 5) in an acute psychiatric unit in Ireland. Following a 12-week implementation of Safewards, three focus groups were conducted, two with nursing staff and one with nurse managers. Data were analysed using Braun and Clarke thematic analysis framework which supported the identification of four themes: introducing Safewards, challenges of Safewards, impact of Safewards and working towards success.

**Results:**

The findings indicate that the process of implementation was inadequate in the training and education of staff, and that poor support from management led to poor staff adherence and acceptance of the Safewards interventions. The reported impact of Safewards on nursing practice and patient experience were mixed. Overall, engagement and implementation under the right conditions are essential for success and while some participants perceived that the interventions already existed in practice, participants agreed Safewards enhanced their communication skills and relationships with patients.

**Conclusion:**

The implementation of Safewards requires effective leadership and support from management, mandatory training for all staff, and the involvement of staff and patients during implementation. Future research should focus on the training and education required for successful implementation of Safewards and explore the impact of Safewards on nursing practice and patient experience.

**Supplementary Information:**

The online version contains supplementary material available at 10.1186/s12912-021-00554-x.

## Background

One of the main functions of acute psychiatric units is to keep patients safe [1] and a lack of safety experienced by patients and staff in psychiatric units is a major concern. Certain behaviours known as conflict events displayed by patients such as aggression and violence, suicidal or self-harming behaviour, drug/alcohol use, absconding, rule-breaking and treatment refusal can threaten patient safety and that of others [2]. A high prevalence of conflict events can have an impact on the unit environment and quality of patient care [3]. Such events can be a challenge for nurses in maintaining their own safety and that of others while also ensuring a safe therapeutic unit environment [4]. While implementing safety and security measures in acute psychiatric units is predominantly the responsibility of nursing staff [4], nursing interventions aimed at improving safety on psychiatric wards have been shown to be ineffective and potentially harmful to patients and staff [5]. Containment methods, which include the use of coerced medication, restraint and seclusion, are frequently used when patients exhibit or direct conflict behaviours towards themselves, others or their surroundings [2]. Consequently, the efficiency of containment methods are often debated in the literature due to lack of evidence regarding their effectiveness [6, 7] and the negative physical and psychological harm they cause to patients and staff [8, 9].

In response to international concerns regarding the potential harm that containment methods can have on patients and staff, efforts have been made to reduce or eliminate their use in psychiatry. Internationally, legislation, proposals and professional guidelines have been developed and introduced to control the use of containment methods to ensure the safety of patients and staff [10–16]. These developments have led to policy changes, practice initiatives and a growing body of research aimed at improving nursing interventions when managing risk [17], with modern acute psychiatric units adopting less containment methods and safer unit environments [18]. This focus on a safer unit environment is relevant as the frequent use of containment methods has not been successful in decreasing conflict [7, 19]. However, other environmental aspects such as; nurse positive attitudes and behaviours [20], positive ward environment [21, 22] and a greater emphasis on creating safer environments through meaningful therapeutic engagement and treatment programmes with patients [5] have been shown as beneficial in preventing incidents of aggression and violence. Furthermore, nursing interventions that enhance nurse-patient relationships promote safety and improve the quality of patient care [5, 23, 24]. Thereby, a comprehensive and proactive approach to managing safety and risk from the perspectives of nursing practice is warranted and the Safewards model supports this approach [20].

The Safewards model [20] provides a comprehensive model for understanding the internal, external and situational-interactional factors that influence conflict and containment in acute psychiatric units by identifying six conflict originating domains; the staff team, physical environment, outside the hospital, the patient community, patient characteristics and the regulatory framework, which can trigger specific flashpoints (e.g. social and psychological situations that signal and precede conflict behaviours), which can ultimately lead to the use of containment methods. The Safewards model recognises patient and staff modifiers which have the capacity to influence rates of conflict and containment, and generated 10 nursing interventions to address the various flashpoints derived from the conflict originating domains. The Safewards model is an evidence-based model that provides effective nursing interventions to create safer therapeutic ward environments [20]. The model highlights how staff can reduce rates of conflict and containment by implementing 10 Safeward interventions: clear mutual expectations, soft words, talk down, positive words, bad news migration, know each other, mutual help meetings, calm down methods, reassurance and discharge messages. These interventions focus nurses to challenge and change their attitudes and behaviours towards patients in order to improve relationships and safety in units [18]. The Safewards model acknowledges that acute psychiatric units can be unsafe and promotes a shared commitment to safety as it advocates nurses and patients collaboratively working together to improve safety in units. Several interventions such as discharge messages, clear mutual expectations and mutual help meetings draw on the capabilities of patients themselves to influence ward culture and safety. The effectiveness of the Safewards interventions was demonstrated in the Safewards cluster randomised controlled trial which reported a 15% reduction in conflict events and a 26.4% reduction in containment methods used [18]. Internationally, the Safewards model has gained increasing acceptance and recognition for its ability to improve safety in units and in 2015, it was referred to as a framework for anticipating and reducing violence and aggression on inpatient psychiatric wards [11]. As a new model there are concerns regarding the rigour of Safewards trials [25] and the fact that few independent evaluations exists of the implementation of the Safewards model [26].

As the Safewards model is relatively new, limited amount of published studies exists regarding its impact. While some studies exist highlighting a reduction in conflict and containment in wards [18, 27, 28]. There is a failure to explore nurses’ experience of Safewards implementation and if Safewards interventions have made any real impact on nursing practice and patient outcomes. This lack of evidence in assessing the implementation of Safewards may in part be due to the fact that such evaluations are heavily dependent on the support and willingness of nursing staff to engage with the interventions on busy wards [29–31]. Furthermore, the high acuity of inpatient units, staff shortages, large numbers of temporary/relief staff, high patient turnovers, critical ward incidents and negative staff attitudes are all seen as impacting implementation [30–32]. Thereby, to successfully implement Safewards interventions and to ensure their sustainability over time, there is a need to investigate and understand the experience of nurses directly involved. This paper reports mental health nurses’ and managers’ experience of the introduction and practice of the Safewards model and three Safewards interventions (reassurance, soft words and discharge messages) in an acute psychiatric unit in Ireland.

## Methods

### Study design

This study used a qualitative descriptive design [33] to identify nurses’ experiences and the factors that support or hinder the implementation of Safewards. A qualitative descriptive research design was found to be the most suitable research design for this study as it enabled a detailed description of the introduction and practice of Safewards from the participants own experience.

### Study setting

The study setting involved a 42-bed acute psychiatric unit in the Mid-West region of Ireland. Focus groups were conducted with 21 registered nurses/nurse managers following a 12-week implementation of three Safewards interventions, reassurance, soft words and discharge messages (Table 1). A phased-in implementation strategy was decided upon for the introduction of the Safewards model and the 10 interventions in the unit over a 12-month period. This phased-in implementation strategy was decided upon so nurses would be provided with continuous support and feedback and that the interventions would be introduced, developed and embedded in a staged manner rather than implementing all 10 interventions together. Nurse managers were appointed as ‘champions/co-champions’ to lead the implementation of Safewards on the unit. A train-the-trainer model was adopted as recommended by the Safewards team [18]. The champions/co-champions attended a two-day local in-service workshop on Safewards prior to implementation. The champions/co-champions provided information and support to staff and their role was to engage staff in the learning materials regarding the Safewards model and interventions. Staff were provided with a resource folder containing information regarding the Safewards model, the three chosen interventions and access to intervention training videos on an office computer. The champions/co-champions developed their unit-based implementation strategy for the three interventions being implemented on the unit. For the intervention “reassurance”, the champions/co-champions reminded staff to implement “reassurance” when needed and to document incidents of “reassurance” in a reassurance book. For “soft words”, the champions/co-champions download posters available from the Safewards website and these were displayed and changed regularly in the nursing office to inform staff. For “discharge messages”, a discharge tree was painted on a wall in the unit and staff offered patients the opportunity to hang messages on leaves of the discharge tree, that were stored in the discharge folder in the office. The study was led by the first author, a registered psychiatric nurse working in the unit as it was envisaged the results would be beneficial for the implementation and practice of the remaining seven Safewards interventions.

**Table 1:** Description of Safewards interventions

Safewards Interventions	Description
Reassurance	Reassuring explanations to all patients following potentially frightening incidents
Soft Words	Short advisory statements outlining potential strategies to use when handling flashpoints (e.g. responding to patient requests or limit setting), which are hung in the nursing office and changed regularly
Discharge Messages	A display of positive messages about the ward from discharged patients

### Ethical approval

Ethical approval was granted from the Health Service Executive Research Ethics Committee for the relevant hospital. Written and verbal information regarding the study were given to participants, and all participants signed a consent form. Participants were informed that confidentiality was a shared responsibility among focus group participants and were asked to sign a declaration of confidentiality to ensure that the discussion and identity of fellow participants would not be disclosed outside the focus group. Participation was voluntary and participants were informed that they could withdraw from the study at any time and that their anonymity was assured.

### Sample

A purposive sample of registered psychiatric nurses/managers (*n* = 56) with over 1 year nursing experience who were employed in the unit during the implementation of Safewards were invited to participate via an information pack containing an invitation letter, information sheet and an expression of interest form (*n* = 48 nurses and *n* = 8 nurse managers). A total of twenty-one (*n* = 16 nurses and *n* = 5 managers) returned the expression of interest form indicating their interest to participate in the focus groups and all participated in the study. The sample included both males and females, ranging in age (22–54) and years of nursing experience (1–35).

### Data collection

Focus groups were conducted to facilitate a discussion among participants about their experiences of the introduction and practice of the Safewards model and three Safewards interventions in the unit. Three focus groups were conducted, two with nursing staff (*n* = 9, *n* = 7) and one with nurse managers (*n* = 5) who were champions/co-champions leading the implementation of Safewards on the unit. Each focus group lasted approximately 90 min and were facilitated in a meeting room in the acute unit. The first author (HL) conducted the focus groups using an interview guide (supplementary file 1), audio-recorded the interview and transcribed verbatim and the third author (TH) verified accuracy of transcription prior to analysis. Participants were afforded the opportunity to review the focus group transcript and make any comments or amendments as appropriate, no feedback or amendments were received.

### Data analysis

Data were analysed using Braun and Clarke six-phase framework for thematic analysis [34]; becoming familiar with the data, generating initial codes, searching for themes, review of themes, defining and naming themes and report writing. Utilising an inductive approach, data was coded and themed by the first and third authors who independently read and reread the transcripts to identify themes. The authors met to discuss and confirm the themes identified, and these were reported in the form of a summary of key themes and subthemes supported by participant illustrative quotes.

## Results

**Fig. 1:**
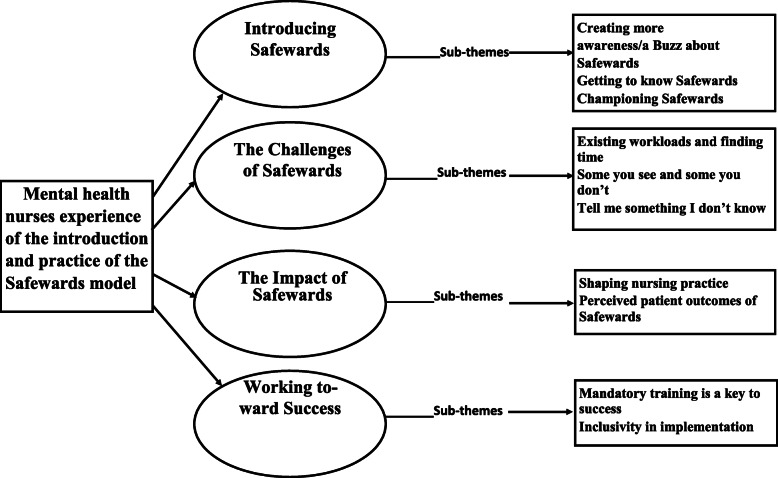
Thematic map

The themes developed from data analysis were; introducing Safewards, the challenges of Safewards, the impact of Safewards and working towards success. The use of subthemes allows for a deeper understand of the key themes (Fig. 1) and within the results, nurses are referred to as ‘nurse participants’ and nurse managers are referred to as ‘champions/co-champions’.

### Introducing Safewards

This theme explored nurse participants and champions/co-champions experiences of how the Safewards model and interventions were introduced in the unit. All participants described how they were initially introduced to Safewards, how they experienced the learning materials for Safewards and the/their role of champions/co-champions. Nurse participants described the process as superficial as Safewards was only briefly introduced and explained to them in handovers and weekly staff meetings and more discussions were warranted for staff to increase staffs’ understanding of the project. However, both nurse participants and champions/co-champions experienced a great ‘buzz’ around the introduction of Safewards however, after the launch, enthusiasm around Safewards started to fade away and die off.

*‘it was discussed at a meeting and then it was briefly introduced and then posters went up but other than that I didn’t hear too much about it in between it wasn’t really explained properly, the enthusiasm just kind of dies out after a while’ (Focus group 2, participant 3).*

For the champions/co-champions they felt that staff perceived the implementation of Safewards as a ‘fleeting sort of a thing’ or an ‘academic exercise’.

One champion felt that staffs’ perception of Safewards was that it was not going to remain on the unit after it had been introduced.

*‘It’s kind of perceived as just something that’s being done . . like we’re going to introduce this and it’s going to come and it’s going to go’ (Focus group 3, participant 1).*

While this perceived barrier existed the champion/co-champions admitted that they should have engaged in ongoing discussions about Safewards to keep the ‘buzz’ going so it remained relevant for all staff.

Many of the nurse participants were not fully informed or aware of the materials introduced for Safewards, such as the reassurance book or discharge folder, which led to incidences where they were not implemented. Nurse participants felt there should have been more discussion and information given about the materials for the interventions in order for them to be more aware of the interventions. They were critical of the learning materials, such as the Safewards folder and videos, suggesting they were unsuitable for staff. Nurse participants also identified the difficulties in finding time to engage with the learning materials in busy work environments and consideration needed to be given to each nurses’ own unique learning styles. It was argued that some people do not learn from reading and the champions/co-champions should have acknowledged that people have different leaning capacities. It was suggested that management should have been more active in the education of staff reducing the necessity for staff to self-education on the Safeward interventions.

*‘when it’s up maybe in a computer and there’s videos we have to go and find information ourselves because it is not being fed to us sometimes when you want to start implementing something you need to be fed the information more’ (Focus group 1, participant 2).*

The champions/co-champions perceived that staffs’ understanding of Safewards was limited as they perceived that staff had either not engaged in the learning materials for Safewards or had forgotten about it. They agreed that the information and materials given to staff could have been an issue and that there should have been a greater emphasis put on education and training for staff when Safewards was introduced.

While nurse participants described how the champions/co-champions facilitated the implementation of Safewards, they did not want to increase their ‘stress’ or further ‘burden’ them for information about Safewards. Nurse participants felt that the champions/co-champions had other responsibilities on the unit and therefore perceived they did not have the time to engage with staff about Safewards. One champion discussed the difficulties of their role as both a manager and a Safewards champion.

*It’s very hard if you are a champion cause you still have a caseload on the ward, you’re still doing all your other jobs and then you have to find time to sit down and talk about this with staff’ (Focus group 3, participant 3).*

Nurse participants felt that they should have been provided the opportunity to be a champion/co-champion as they perceived that this would inform and engage them more with Safewards.

*‘that goes back on the champions or co-champions again who has a busy caseload, it’s not fair on them that they got to take an hour out of their day every day that they’re on duty to speak to the staff’ (Focus group 2, participant 1).*

The champions/co-champions agreed that their role was to inform staff about the Safewards model and encourage staff to implement the interventions. They felt that they should have had regular meetings amongst themselves to identify any issues and to provide support. They agreed that they needed to ‘reflect’ on their roles as champions/co-champions.

### The challenges of Safewards

This theme explains the challenges of implementing Safewards on the unit. A challenge clearly articulated by the nurse participants was that of feeing overworked and understaffed. This feeling was reinforced by the busy nature of an acute psychiatric unit and their existing workloads. However, it was the difficulty of finding the time to implement the Safewards interventions as the real challenge expressed.

*‘the ward is so busy and you’ve a caseload, you’ve meetings during the week you’ve how many specials now and you’re trying to go on your own break, cover someone else’s when you come back, do ward rounds, do pharmacy and then finding the time for this stuff in between’ (Focus Group 2, participant 3).*

The champions/co-champions acknowledged that staff felt overworked on the unit and how they could have perceived the implementation of Safewards as an additional workload.

*‘you are already feeling overworked and then somebody is asking you to refer to this it feels like it’s more of a workload (Focus group 3, participant 5).*

All participants described how the interventions “reassurance” and “soft words” lacked visibility in comparison to the intervention “discharge message” and therefore they could not tell if they were being implemented on the unit. Nurse participants described the visible presence of Safewards on the unit (e.g. soft words posters, reassurance book, discharge book and discharge tree) but had difficulty in describing how they implemented the interventions "soft words" and "reassurance" in practice which may suggest that they may have been implemented without staff having any real understanding of these interventions and how they should be implemented.

*‘the other two ("reassurance" "soft words") you weren’t recognising as much that you implemented them but you see you’ve done something when you implement this one ("discharge messages") so you like physically hand someone something and they put it up on a wall so it’s like you’ve done an action whereas the other two its verbal’ (Focus group 2, participant 5).*

Nurse participants felt that the Safewards interventions “reassurance” and “soft words” already existed in their nursing practice which created ‘negative vibes’ about Safewards amongst staff. This feeling arose due to the fact that nurses felt interventions for example, "soft words" was already part of their practice and that they are always ‘professional’ and ‘mindful’ when they spoke to patients. Nurse participants felt that their existing nursing practice was being questioned when they were told to implement the intervention "reassurance”, something they perceived that they were implementing as part of their ‘nursing degree and training’.

*‘it’s also putting nurses backs up straight away as you are like that’s my job, I do reassurance I don’t need to be told and then to be implemented in such a big way it’s like you’re saying are we not reassuring people’ (Focus group 2, participant 5).*

However, the champions/co-champions argued that if staff had engaged in the learning materials for the intervention "reassurance" they would have understood the difference between a structured reassurance process and reassurance in their daily practice.

*‘The training on the desktop it’s very clear when that structured reassurance should be given so if everyone did that training then it’s very obvious how it should be implemented’ (Focus group 3, participant 2).*

The champions/co-champions identified negative staff attitudes as a major challenge when implementing Safewards. They felt staff became defensive during the implementation of Safewards as they perceived that they were doing something ‘wrong’ and that management were now telling them what to do.

### The impact of Safewards

This theme explored the experience of how Safewards impacted nursing practice and patient experience on the unit. In relation to nursing practice, there was a mixed response from nurse participants, where some perceived it had no change to their nursing practice or patient outcomes, others felt it improved their communication skills and relationships with patients. Most nurse participants, perceived Safewards as having no impact on their nursing practice, as they felt they were already implementing the interventions “reassurance” and “soft words” and therefore had not practiced any differently since Safewards was introduced. Despite support for the intervention "discharge messages", nurse participants perceived that it was not ‘common practice’ for it to be implemented which resulted it being abandoned by staff. Some nurse participants were critical of Safewards being introduced to the unit as they felt there was no need to change their current practice and perceived Safewards was ‘only going back over what we already know and what they we were already doing’.

*‘whether there is massive impact or not I’m not really sure because we would have been doing most of the stuff previously anyway like it would have been part of our nursing practice (Focus group 1, participant 6).*

While many felt that Safewards had not impacted their nursing practice, some nurse participants described how Safewards made them more aware and more mindful of how they spoke and cared for patients.

*‘It enhanced my awareness when I am talking to a patient, you’re aware Safewards is going on and you’re trying to make a bit more of an effort when you’re talking to patients’ (Focus group 2, participant 2).*

Nurse participants described how they have been making more of an effort with patients to be more ‘positive’ and ‘empathetic’ in their nursing approach.

*‘I imagine we are more approachable, like patients know they can ask us something and we will try our best, nothing is out of the way, that we would try help them’ (Focus group 1, participant 8).*

Nurse participants demonstrated an increased understanding of patient behaviour which led them to providing explanations to patients when they had to give reasons for saying no. One nurse participant provided an example of how implementing the intervention “soft words” improved communication and relationships between nurses and patients

*‘I think the relationship between the patient and the nurse is better cause when you give a reason for saying that you can’t do something now once they get the reason they appreciate it a lot more than just saying no’ (Focus group 1, participant 4).*

Similarly, the champions/co-champions described how the implementation of Safewards had a positive impact on nursing practice as they felt that the interventions made staff ‘more mindful’ of their practice and more ‘approachable’ to patients.

One champion described how "soft words" improved nurse-patient relationships on the unit.

*‘it’s made them more aware on how they talk to patients . . .. I imagine it’s improved the relationships that nurses’ develop with patients here’ (Focus group 3, participant 2).*

While some nurse participants perceived that Safewards would have a positive impact on patient experience, others argued that it would not be a ‘defining factor’ that would impact their inpatient experience. Nurse participants described how it was difficult to measure the impact that Safewards would have on patients due to high patient turnover. They also perceived that patients were not aware of Safewards being implemented and that the booklet given on admission was not informative.

One nurse participant described how patients did not know about Safewards being implemented on the unit.

*‘You wonder do some of them even know about it, they probably do not even read those booklets and leaflets about Safewards , I don’t think they know about the unit implementing it’ (Focus group 1, participant 3).*

However, nurse participants felt that the intervention "discharge messages" was visible and accessible to patients and believed they benefited from reading messages of hope and recovery on admission. Similarly, the champion/co-champions discussed the positive impact that the intervention "discharge messages" has on patients’ experience. The champions/so-champions felt that the intervention helped ‘reduce stigma’ for patients being admitted to the unit and supported them to work towards recovery and discharge. Some nurse participants perceived that the interventions "reassurance" and "soft words" already existed in their practice and theyt had not practiced any differently since Safewards was introduced, therefore they perceived that patient outcomes remained unchanged.

*‘Our nursing practice hasn’t changed like we’re not doing anything different than we were before so the patient outcomes are pretty much probably the same as they were prior’ (Focus group 2, participant 5).*

### Working toward success

This theme explores suggestions for the implementation of Safewards on the unit. Nurse participants gave recommendations on how the remaining interventions should be successfully implemented. The need for more in-depth training and education was discussed by all throughout the focus groups and mandatory training was viewed as important for the successful implementation of Safewards. Nurse participants had not received formal training on Safewards and recommended that there should be mandatory training on Safewards for all staff. Engagement in the learning materials for Safewards was optional for staff and nurse participants perceived that mandatory training would be more beneficial as this would ensure that all staff are aware and informed of the interventions being implemented. It would also address concerns that they had about staff turnovers/shift patterns and identify staff who had not received training. Nurse participants perceived that it would highlight the importance of Safewards on the unit and that it would help incorporate the interventions into their everyday nursing practice

*‘if it was done by a training day it would seem to highlight that it is more valuable or a greater deal of importance like if it’s mandatory then it’s essential, it’s an important part of our practice’ (Focus group 1, participant 2).*

Similarly, the champions/co-champions felt that the learning materials provided were inadequate to train/educate staff as they were described as ‘too broad’ and needed to be ‘more specific’. The champions/co-champions felt that staff needed to understand the theory underpinning the model and the potential impact on practice. They described how there was no ‘buy in’ by staff as they were not given enough information on Safewards and that there would be better “buy in” if they were trained.

*‘I think staff nurses should get full training in Safewards as I don’t think they know enough about it, it would give staff a better understanding of it and I think it would help them towards using Safewards better’ (Focus group 3, participant 3).*

There was uncertainty among the champions/co-champions on how the training should be effectively delivered to staff. Similarly, the champions/co-champions perceived that they had not received formal training on Safewards. Although they had attended an informative two-day in service workshop on Safewards prior to implementation, they felt that they were not provided with the necessary skills and strategies to train staff. The champions/co-champions felt that they would need more support and intensive training in order to train staff. It was evident that informal discussions about Safewards with staff had not adequately educated, trained and engaged staff, and formal training delivered by champions/co-champions may have been more effective.

Nurse participants expressed the need to be more involved in the implementation of Safewards as they perceived they were responsible for implementing the interventions. Nurse participants had many suggestions for improving the implementation of the three Safewards interventions, while also having concerns how the remaining interventions would be introduced. Nurse participants discussed the importance of all staff’s opinions during implementation and felt that their views and opinions should be considered by management for Safewards to be effectively implemented.

‘*there has to be emphasis on the importance of the nurses’ opinion before something is brought in, nurses have to be asked what they think of the next stage before is implemented and not just be told when it comes in that this is going to be the norm now and this is what you’ve to be doing, we all feel we have to be asked if we are going to be involved in it (Focus group 2, participant 2).*

The champions/co-champions agreed that staff need to be more involved in the implementation process as they felt that successful implementation of Safewards requires a ‘buy in’ by staff. Participants recommended that a successful implementation of Safewards also requires patient involvement. Staff recommended that management should facilitate Safewards groups with patients, so they are knowledgeable and aware of the Safewards model on the unit and to support them to effectively feedback on the programme. Recommendations were made by nurse participants to undergo patient evaluations of the implementation of Safewards.

## Discussion

The findings of this study indicate the process of implementation was inadequate to train and educate staff. There was no formal training on Safewards which led to staff only having a limited understanding of the Safewards model and interventions. The learning materials for Safewards were heavily criticised by staff who felt it was their responsibility to educate themselves about Safewards. Many staff did not engage with or fully understand the information given which could suggest that the learning materials were unsuitable for staff. A lack of training and education is associated with poor staff adherence and acceptance of the Safewards model and interventions [29]. This finding adds to previous literature which identified that staff only had a superficial understanding of the Safewards model, suggesting that the process of implementation was inadequate to train and educate staff with the necessary knowledge and skills to implement the Safewards interventions [31, 35].

There is limited research available regarding training for Safewards with similar studies adopting the train-the-trainer approach as recommended by the Safewards team [29, 31]. In line with the findings of these studies, it is questionable if the train-the-trainer approach is effective in implementing the Safewards model and interventions. It was found that the champions/co-champions were not discussing Safewards regularly with staff despite their role in providing information to them. Interestingly, staff felt they could not approach the champions/co-champions about Safewards as they felt that it would add ‘stress’ to their existing workload which would demonstrate that staff were unclear about the role of the champions/co-champions. The champions/co-champions in this study perceived that they did not receive formal training in Safewards and therefore felt they were not equipped to train and educate staff. O’Donnell and Boyle [36] found that management need to not only understand the information themselves but be able to communicate information effectively with staff. The champions/co-champions had managerial roles which may have impeded them carrying out their role. Furthermore, staff may have been reluctant to approach their managers with their concerns or issues regarding Safewards, which could have created a disconnect between the champions/co-champions and staff. The champions/co-champions should be selected based on their attitude, motivation and commitment and this study similar to Kipping et al’s [37] highlights that consideration should be given to front line staff in this role to support staff adherence and acceptance.

The findings illustrate a lack of support for Safewards after it was introduced. It was found that Safewards is at risk of losing its momentum if it not supported by management and staff. Previous studies have found that once Safewards was introduced, staff slowly reverted to old practices [30, 32] and that staff often view evidence-based practices as passing ‘fads’ that will soon be replaced with something else [38]. This was addressed by the champions/co-champions who felt that staff perceived the implementation of Safewards as ‘fleeting’ and that it was not going to remain after it was introduced. Like other studies, staff perceive management to have a significant role in promoting and supporting the implementation of Safewards [29, 31]. Staff depicted a lack of support by management during the implementation of Safewards as they were not discussing Safewards regularly with staff. It is the responsibility of management to foster change amongst staff by providing ongoing support, supervision and role modelling during implementation [31].

This study reported existing workload and time constraints, lack of visibility and staff attitudes as challenges during the implementation of Safewards. Staff highlighted the immense pressure of working in an acute unit and had concerns about additional workload and demands when implementing the interventions. A limitation in previous studies was underestimating the impact that the ward environment had on implementation efforts by staff [30, 31]. In recent decades, nurses have been criticised for their lack of time to engage with patients when implementing interventions [39–41]. Staff shortages and task-orientated practices make it difficult for staff to implement the Safewards interventions [29]. Although no suggestions were made on how to reduce the impact of the unit environment on implementation, it has been found that changes to work practices, such as hiring more staff and freeing up more staff time could allow staff more time to implement new interventions [24].

It was found that the interventions “reassurance” and “soft words” lacked visibility in comparison to “discharge messages”, making it difficult to measure the degree to which staff engaged with these interventions. Bowers et al. [18] acknowledged the difficulty in measuring staff adherence to the Safewards interventions as they are targeted to change interactions between staff and patients. In previous studies, staff adherence to the interventions was measured using the Safewards fidelity checklist [18, 27]. A limitation of the study is the failure to use the Safewards fidelity checklist as the implementation of Safewards began before the study was commenced. However, it is important to note that the checklist measures the visible evidence of the interventions on display rather than the degree to which staff engaged with or used the interventions [18]. This was evident in the findings where staff described the visible presence of Safewards on the unit but were unable to describe how they implemented “reassurance” and “soft words” in practice. This may suggest that these interventions may have been implemented without staff having any real understanding of the interventions and how they should be implemented. The study illustrates the need for good quality evaluations during implementation to measure staff adherence.

Negative staff attitudes pose a significant barrier to the implementation of Safewards. Staff believed that the Safewards interventions, “reassurance” and “soft words” already existed in their nursing practice which created a ‘negative vibe’ among staff. It has been argued that the Safewards model represents evidence-based practice in mental health with many of the interventions developed out of existing good practice identified in research [29]. Higgins et al. [31] found that staff felt their existing nursing practice was being criticised and that they were not being recognised for their existing knowledge and skills. This was evident in the findings as staff felt that their existing nursing practice was being questioned. The implementation of evidence-based practice is effective when it supports the values, beliefs and needs of staff who prefer to acquire new skills consistent with practices that they feel requires change [42]. While staff are familiar with some aspects of Safewards, reluctance or negative attitudes may have their origin in a poor understanding of the principles and process of Safewards. In the online Safewards training package developed by Bowers [43], it identifies what the interventions are not in order to differentiate the interventions from existing practices. However, poor staff engagement in the online training videos for Safewards may have led to misunderstandings and assumptions being made about the interventions by staff. This was addressed by the champion/co-champions of the study who felt that if staff had engaged in the learning materials for Safewards, they would have had a greater understanding of what the interventions were about and how they differentiated from existing practices. However, while the champion/co-champions perceived poor engagement they did not articulate any measures to encourage, support or reinforce engagement. Although this is needed to be considered in light of the fact that the champion/co-champions were leading out on this initiative in addition to their normal role and responsibilities. Raising the question as to how we support the implementation of new practice in busy work environments and the need for stakeholder buy-in and support.

When exploring the impact of the Safewards interventions had on nursing practice and patient experience in the study, there was a mixed response from staff. For some staff, the interventions already existed in practice and therefore believed they had no impact on nursing practice or patient outcomes. These findings expand on James et al. [30] study where staff felt that the underlying philosophy of the Safewards model was something they already learnt in practice and that some of the interventions were a replicate of what staff were already doing. Staff engagement during implementation is not only determined by the knowledge and skills of staff but whether the values underpinning Safewards fit with their personal beliefs about their nursing practice and the potential impact that it could have on it. It has been found that staff were pessimistic about the potential effect that the Safewards interventions would have on efforts to reduce aggression and violence [29]. This suggests that there was an emphasis on Safewards being a conflict and containment model rather than a model that enhances safety through improved nurse-patient relationships and collaborative practices [20].

Despite the above findings, some staff spoke positively about the Safewards interventions and how they enhanced their communication skills and relationships with patients. Staff were more mindful of how they spoke and cared for patients which made them more ‘empathic’ and ‘positive’ in their nursing approach. Exploring patient experience and outcomes is identified as an important factor when implementing new practices and interventions in healthcare [44]. One study that explored patients’ perceptions and experiences of Safewards found that patients valued the implementation of Safewards as it created a feeling of respect and understanding between patients and staff [32]. The failure to include the experiences of patients in evaluations of Safewards is an acknowledged limitation of the study.

Staff had recommendations for the implementation of the remaining Safewards interventions which indicates that they would be willing to engage in the implementation process under the right conditions to ensure success. The need for more extensive training and education was echoed throughout the study. Staff engagement in the learning materials for Safewards was optional and such an approach was considered a weakness of the programme. In hindsight, it was felt that mandatory training indicates organisational commitment to ensure a greater deal of understanding, importance and acceptance of Safewards by staff. Staff suggested that they would be more willing to engage with the Safewards interventions if they were given the opportunity to attend training before implementation. The need for mandatory training was also addressed by the champions/co-champions who felt there would be a better ‘buy in’ if staff were trained. They also expressed concern that there was not enough emphasis on education and training during implementation as staff had a ‘limited’ understanding of the Safewards model. These findings resonate with James et al. [30] study where staff struggled to understand the theory or function underpinning the Safewards model and interventions. Training and education should focus on the theory underpinning the model and rational for the interventions This may help address the negative attitudes and scepticism of staff and encourage their adherence and acceptance of the interventions. While the champions/co-champions supported staff training for Safewards, there was uncertainty on how this training should be provided to staff. The champions/co-champions perceived that they had not received formal training on Safewards and suggested how they would have to undergo more intensive training in order to train staff. Previous studies have recommended that training not only be provided to front line staff, but also to managers who have an important role in engaging, motivating and educating staff [31, 32]. In line with Higgins et al. [31], there needs to be a review of the learning materials for Safewards so that they are suitable to staffs’ level of knowledge, skill and expertise. This would ensure that the materials are adhered to and understood by staff.

The successful implementation of Safewards requires the commitment of all staff [29, 30] as the lack of involvement of staff is a well-known barrier to the successful implementation of new practices and initiatives [45]. In this study staff expressed the need to be more involved in the implementation of Safewards as they felt they were responsible for implementing the interventions. The champions/co-champions in this study described how a successful implementation of Safewards requires a ‘buy in’ from staff and that introducing Safewards requires a whole team approach where everyone is implementing Safewards together. Staff felt that it was important that their opinion and views be considered during implementation. This highlights the need for a co-created implementation strategy, that engages staff in the implementation process to enhance adherence and acceptance of Safewards [37]. It is also recognised that within this created strategy or plan that patient involvement is important [37] as poor patient involvement can impact staff adherence and acceptance of the Safewards interventions [29, 30]. Patient feedback encouraged implementation therefore there should be ongoing discussions with patients to allow feedback on Safewards [30].

## Conclusion

While mental health service provision has advanced and the majority of people with mental health problems can be treated in the community, inpatient psychiatric care remains necessary for a small group of patients who cannot be treated safely or effectively at home [31]. Many of these patients have acute symptoms, can be a danger to themselves and/or others resulting in aggression, violence and conflict [41]. An important factor is limiting the likelihood of aggression, violence and conflict through the therapeutic alliance between nursing staff and patients [46, 47]. This paper provides an insight into mental health nurses and champions/co-champions experiences of the introduction and practice of three Safeward interventions, adding to the scant qualitative research available on the experiences of those directly involved. The study highlighted the difficulty in implementing Safewards in a busy acute mental health unit and that a lack of training and education led staff to only superficially understand the Safewards model and interventions, suggesting that the process of implementation was inadequate. Management have an important role in supporting and engaging staff during the implementation of Safewards, through effective leadership, support, active involvement, supervision and facilitating regular discussions with staff. Successful implementation requires consideration be given to pre-existing knowledge, skills and attitudes, time available to staff to engage with interventions and these should be considered in terms of the ward environment, staffing levels/shortages, additional workload and time constraints have on implementation efforts of staff.

Although there was a mixed response on the impact of Safewards in this study on nursing practice and patient experience, the positive impact that Safewards can have on nursing practice and nurse-patient relationships emerged from the study. It has been found that the Safewards interventions enhanced communication with patients, which improves relationships with patients [27, 48], promotes a shared commitment to safety through collaboratively working to improve safety [24, 49]. The study highlights the need for mandatory training for all staff, effective leadership and support by management and the involvement of staff and patients during implementation. Future research should focus on the training required for Safewards and explore the impact of Safewards on nursing practice and patient experience.

## Supplementary Information

**Additional file 1: Supplementary file 1.** Focus Group Interview Guide.

## Data Availability

Data will not be shared due to difficulties in the anonymization of qualitative data as participants within this study shared their views and experiences with the assurance that their confidentiality and anonymity would be protected. Hence, the research data is not available publicly because this would compromise individual privacy and our ethical approval conditions.
