# Supplementary material for: Mental health nurses experience of the introduction and practice of the Safewards model: a qualitative descriptive study
Source: BMC Nurs. 2021 Mar 11;20:41. doi: 10.1186/s12912-021-00554-x (PMC7953680; doi:10.1186/s12912-021-00554-x)
Supplement: Supplementary file 1 — Additional file 1: Supplementary file 1. Focus Group Interview Guide. [file 12912_2021_554_MOESM1_ESM.docx]

# Additional file 1 Focus Group Interview Guide

# Mental health nurses experience of the introduction and practice of the Safewards model: A qualitative descriptive study

**Authors and Co-authors**

Heather Lee, Owen Doody, Therese Hennessy.

**Corresponding author**

Therese Hennessy, MSc, BSc, RPN. Lecturer, Department of Nursing and Midwifery, University of Limerick, Limerick, Ireland. [therese.hennessy@ul.ie](mailto:therese.hennessy@ul.ie), +35361213365.

**Authors**

Heather Lee, Msc. BSc. RPN, Staff nurse, Mid-West Health Service Executive, Limerick, Ireland.

Owen Doody, PhD. MSc. BSc. RNID. Senior Lecturer, Health Research Institute, Department of Nursing and Midwifery, University of Limerick, Limerick, Ireland.

**Additional file 1:**

Word Document

**Focus Group Interview Guide**

Introduction

- Welcome Group: Provide an overview of the research study and research process
- Declaration of Confidentiality
- Consent form
- Ground rules for focus group
- Answer any questions that participants may have before the focus group discussion.

Questions for Focus Group

1. Can you tell me about your experience of the introduction of the Safewards model on the unit?
2. *Now I want to talk about your experiences of the Safewards intervention Reassurance in practice:*
3. What were the factors that you experienced that helped facilitate the Safewards intervention *Reassurance* in practice?
4. What were the barriers that you experienced when implementing the Safewards intervention *Reassurance* in practice?
5. In your opinion, how has the Safewards intervention *Reassurance* impacted your nursing practice?
6. *Now I want to talk about your experience of the Safewards intervention Soft Words in practice:*
7. What were the factors that you experienced that helped facilitate the Safewards intervention *Soft Words* in practice?
8. What were the barriers that you experienced when implementing the Safewards intervention *Soft Words* in practice?
9. In your opinion, how has the Safewards intervention *Soft Words* impacted your nursing practice?
10. *Now I want to talk about your experience of the Safewards intervention Discharge Messages in practice:*
11. What were the factors that you experienced that helped facilitate the Safewards intervention *Discharge Messages* in practice?
12. What were the barriers that you experienced when implementing the Safewards intervention *Discharge Messages* in practice?
13. In your opinion, how has the Safewards intervention *Discharge Messages* impacted your nursing practice?
14. In your own opinion, how has the Safewards model impacted patient experience on the unit?
15. Is there anything that anyone would like to say about the Safewards model practiced on the unit that we have not already talked about?

*Thank you all for your participation in this focus group.*
